# Ram semen preserved at 0°C with soybean lecithin Tris-based extender substituted for egg yolk

**DOI:** 10.5713/ajas.20.0118

**Published:** 2020-05-12

**Authors:** Jian-qing Zhao, Guo-liang Xiao, Wen-liang Zhu, Di Fang, Na Li, Chun-mei Han, Qing-hua Gao

**Affiliations:** 1 College of Life Sciences, Tarim University, Alar, Xinjiang 843300, China; 2 Key Laboratory of Tarim Animal Husbandry Science and Technology, Xinjiang Production & Construction Corps, Alar, Xinjiang 843300, China; 3 Kashgar Animal Husbandry Workstation, Kashi, Xinjiang 844000, China

**Keywords:** Ram, Semen, Diluent, Soy Lecithin, Egg Yolk

## Abstract

**Objective:**

The present study evaluated the preservation of ram semen at 0°C using soybean lecithin with a Tris-fructose extender.

**Methods:**

Semen was collected by artificial vagina ejaculation from six rams with proven fertility. High quality ejaculates were diluted by soybean lecithin (0.25%, 0.5%, 0.75%, 1.0%, 1.25%) using Tris-fructose extender and control (Tris-fructose egg yolk extender), respectively. The ejaculates were diluted to a concentration of 5×10^8^ sperm/mL, followed by cooling to 0°C in 90 min and maintaining the temperature for 12 days. The diluted semen samples were examined and recorded for sperm progressive motility, acrosome integrity at 0, 24, 72, 144, 216, 288 h, respectively. Two hundred and twenty-three ewes were inseminated for 216 h with optimal soybean lecithin concentrated semen or control via trans-cervical insemination.

**Results:**

The results showed that there were no differences in sperm progressive motility at 0, 24, 72, and 144 h (p>0.05). After 216 h, the sperm progressive motility in the control group and 0.5% concentration groups was significantly higher when compared to 0.25% concentration (p<0.05). The 0.5% concentration group demonstrated the highest survival rate and had no difference with the control group (p>0.05). At 216 h, the sperm progressive motility of all groups was still above 50%. The acrosome integrity of all groups was decreased with prolongation of storage time, but there was no difference at each time point (p>0.05). There was no significant difference in the lambing rate and pregnancy rate between the 0.5% concentration group and the control group (p>0.05).

**Conclusion:**

These results suggest that ram sperm is capable of fertilization after preservation at 0°C with 0.5% of soybean lecithin in Tris-based extender substituted for egg yolk and produce normal offspring after insemination.

## INTRODUCTION

Long-term preservation of sperm in the field of animal and human medicine is regarded as an important approach for enhancing reproductive technology [1]. Since the invention of freezing technology, the sheep sperm freezing technology has not made substantial progress, and the reason for this is that the sheep sperm is vulnerable to cold shock, and cryopreservation of the sperm seriously reduces the motility of the sperm. The membrane function and the biology of sperm cells are affected, which thus reduces its ability to bind to the oocytes [2,3]. Therefore, improved techniques for long-term preservation of sheep’s semen are of great importance.

Researchers have been keen on using yolk as a cryoprotectant for preservation of animal semen. However, yolk is an animal-derived substance, and it might increase the pathogenic microorganisms during the process of transportation, producing substances that are not conducive to sperm survival. Also, there is no standardized operation that preserves the sperm. Researchers have been actively looking for new substances to replace yolk [4–6]. In addition, the yolk of the birds is more vulnerable to pathogens and might spread diseases during transportation in various countries. Therefore, the harm of using yolk as cryoprotectant should be avoided [7].

Recently, scientists are working on uncovering a new sperm protective agent that replaces yolk. It is necessary to effectively avoid the addition of animal-derived substances, and soybean lecithin (SL), which is a plant source, is currently being investigated [8]. It not only prevents the occurrence of health risks, but also improves the efficiency of semen preservation [9,10]. Recent studies revealed that SL assists in freezing bovine semen [11], ram [12,13], and goat [14].

SL is considered as a valuable source of plant-based phospholipids in commercial extenders that are used for freezing mammalian sperm without clearing the levels and adjustments due to trade protection. The Tris-fructose egg yolk dilution in our laboratory has a stable effect. The egg yolk in the dilution is replaced with soybean lecithin in equal amounts, and other components and factors remain unchanged. The content of lecithin in the yolk is about 10%, and the content of yolk in the diluent of Tris-fructose egg yolk is about 23%. The content of lecithin is converted to diluent by about 3%. The concentration of 1.25% to 10% SL was used instead of egg yolk to preserve the sheep sperm, and the effect of 1.25% concentration was considered much better, However, it was not determined whether adding soybean lecithin with concentration less than 1.25% had better effect [15]. Therefore, this study was designed to investigate the effect of 5 concentrations (0.25%, 0.5%, 0.75%, 1.0%, and 1.25%) of SL as an alternative for egg yolk in ram semen as an extender of sperm quality.

## MATERIALS AND METHODS

### Ethics statement

All animal experiments were conducted according to the Regulations and Guidelines for Experimental Animals established by the Ministry of Science and Technology (Beijing, China, revised in 2004) and the study was approved by the Institutional Animal Care and Use Committee of Tarim University.

### Animals and semen collection

All experiments were performed in the Tarim University (40°54′ N, 81°30′ E) of Xinjiang of China. Semen samples obtained from six mature Duolang rams (2 to 3 years of age) were used in this study. The physical and sexual desire of these rams remained normal and they were capable of fertilizing and producing offspring through artificial insemination (AI). During the experiment, they were fed and managed uniformly. Rams were kept in a wide playground and had free access to water, conventionally. Carrots and eggs were fed 40 days before the experiment, and exercise was strengthened. From each ram, ejaculates were collected using artificial vagina ejaculation as described by Quinn et al [16]. The semen was collected from 18 ejaculates of six rams and was made into 3 test samples for each group.

### Semen processing

After semen collection, the semen was transferred to the isothermal test tube immediately. The test tube was placed in insulation buckets at 37°C and sent back to the laboratory for evaluation in 30 min. Semen evaluation was carried out in accordance to the following criteria and was included in the study after meeting the criteria: ejaculation volume of 1 to 2 mL; sperm concentration of 1.5 to 2×10^9^ sperm per mL; the percentage of progressive motile sperms should be higher than 80% and the percentage of abnormal sperm should be less than 10% for this experiment. Non-conforming semen samples were discarded. The semen samples were pooled to eliminate individual differences and divided into six equal aliquots and kept at 37°C in a water bath. After primary observation, the semen samples were diluted at a ratio of 1:4 (semen:diluent) at 37°C with different amounts of SL (0.25%, 0.5%, 0.75%, 1.0%, 1.25%) and control (23% egg yolk) in each group. The samples were placed in a refrigerator that was cooled to 0°C for 90 mins, and then ice cubes were added to the water bath for long-term storage of diluted semen for inspection.

The basic solution formula of Tris diluent: double distilled water (136 g), Tris (4.84 g), fructose (2.0 g), citric acid (2.68 g), adenosine triphosphate (100 mg), bovine serum albumin (300 mg), vitamin C (600 mg), penicillin (400,000 IU) and streptomycin (200,000 IU).

### Determination of sperm progressive motility

The sperm progressive motility was measured at 0, 24, 72, 144, 216, and 288 h after cooling. The semen was quickly and lightly mixed in the refrigerator, 10 μL semen dropped on the preheated slide, covered the slide, placed at 37°C hot table and detected the total number of sperm (≥200 sperm) in three visual fields at 400× under a microscope, and the number of sperms moving in a straight line were observed and counted at 37°C.

$$\begin{array}{l} \text{The sperm progressive motility }(\%)=\text{number of sperm in}\\ \text{straight forward movement}/\text{total number of sperm}\times 100\% \end{array}$$

### Determination of acrosome integrity

The sperm acrosome integrity was measured at 0, 24, 72, 144, 216, and 288 h after cooling. The sperm acrosome integrity was detected by a smear made from a drop of semen on a sterilized slide, followed by fixing with 95% ethanol for 30 minutes, staining with hematoxylin for 20 minutes, and then rinsing with distilled water. After staining with Eosin staining solution for 5 minutes, the purified water was washed and air-dried naturally. The stain was examined under a (400×) microscope. The total number of sperms in different fields (≥200 sperm) and the number of intact sperms are counted.

Sperm acrosome integrity rate (%)=acrosome intact sperm count/sperm count×100%

### Artificial insemination

After the optimal SL concentration group was evaluated in the above experiment, the semen was collected again and stored in the control group and the optimal SL concentration group to prepare for AI. Duolang ewes (n = 223) housed in a free pasture were used for trans-cervical insemination. Ewes were synchronized with a Controlled Internal Drug Release for 14 days. Fifty-eight hours before AI, the sponges were removed, and animals were given an intramuscular injection of 500 to 600 IU of equine chorionic gonadotropin. Each ewe received two semen samples and saved till the ninth day of semen.

Ewes were randomly assigned to the control group (n = 112, ewes inseminated with control diluent) and the optimal SL concentration group (n = 111, ewes inseminated with the optimal SL diluent). Semen from the six rams were randomly injected to ewes and equally distributed for AI. Pregnancy was detected by B-mode ultrasonography on day 40. Pregnancy and lambing rate were recorded as an index of fertility and reproductive performance.

$$\begin{array}{l} \text{Pregnancy rate }=\text{number of gravid ewes}/\text{total number of inseminated ewes}\times 100\%\\ \text{Lambing rate }=\text{number of lambing ewes}/\text{total number of inseminated ewes}\times 100\% \end{array}$$

### Statistical analysis

The sperm progressive motility rate, and acrosome integrity rate were presented as means±standard error of mean. The data were analyzed using analysis of variance and followed by Tukey’s HSD test to compare the differences between treatments (SPSS software; SPSS, Inc., Chicago, IL, USA). The pregnancy rate and the lambing rate were tested via Chi-square. The significance level of 5% was considered in all statistical analyses.

## RESULTS

### The influence of soybean lecithin on the parameters of semen progressive motility, collected from Duolang rams

The sperm progressive motility showed no significant change with increasing SL dilution concentration at 0, 24, 72, and 144 h. However, after 216 h, the sperm progressive motility in the control group and 0.5% concentration groups was significantly higher when compared to 0.25% concentration (p<0.05), and the 0.5% concentration group had the best sperm progressive motility. There were no significant differences between the 0.5% concentration group and the control group. The progressive motility of these dilutions of ram sperm can still be higher than 50% at 216 h (Table 1).

### The effects of the presence of soybean lecithin in the extender for sperm acrosome integrity

**Figure 1:**
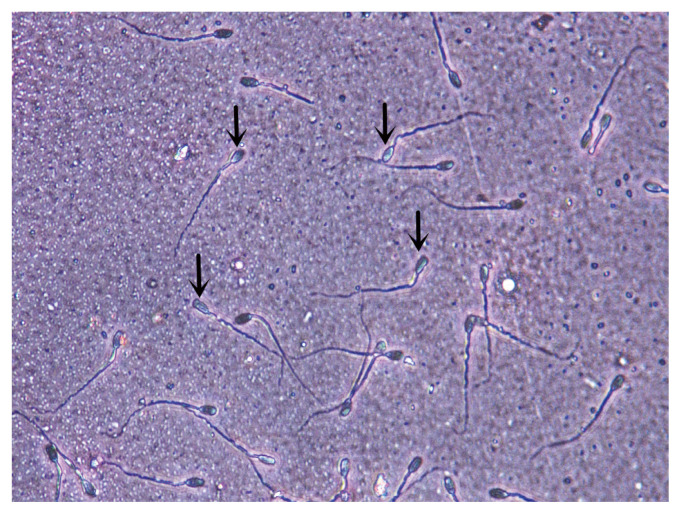
The sperm acrosome integrity image was taken at 144 h, in which the arrow marked sperm is acrosome intact. Sperm acrosome integrity rate were imaged at a magnification of 400× (Hematoxylin and eosin dyeing).

At 0, 24, 72, 144, 216, and 288 h time periods, the sperm acrosome integrity rate of control group was not significantly different from that of the other groups (p>0.05), and the acrosome integrity rate of each group showed a downward trend with the prolongation of time (Table 2), but did not show significant changes with increasing dilution of SL (Figure 1).

### The effects of the presence of soybean lecithin in the extender on pregnancy rate and lambing rate of artificial insemination of semen preserved to 216 h

The control group semen was preserved at 0°C. At 216 h, 112 ewes were artificially inseminated with control groups, in which 74 were pregnant, and the pregnancy rate was 66.1%. The 111 ewes artificially inseminated in 0.5% SL group showed that 72 were pregnant, with a pregnancy rate of 64.9%. Therefore, addition of SL and control in the dilution showed no significant effect on the pregnancy rate of sheep (p>0.05). There was no significant difference between the control group (63.4%) and the 0.5% concentration group (64.0%) on the lambing rate of sheep (Table 3).

## DISCUSSION

The results showed that there was no difference in the sperm progressive motility, acrosome integrity, pregnancy rate and lambing rate between egg yolk dilution solution and when 0.5% SL was used to replace egg yolk for semen preservation. Therefore, 0.5% SL can replace egg yolk for long-term preservation of ram semen at 0°C.

More and more researchers have begun to study the cryopreservation of semen without animal-derived additives [17–19]. Our previous studies have shown that the concentration of 1.25% to 10% SL can be used instead of egg yolk to preserve the sheep sperm, and the effect of 1.25% concentration showed better results [15]. The current study revealed that 0.5% of SL can replace the egg yolk in the Tris dilution, so that the sheep sperm can be stored at 0°C for a longer time. It can be concluded from the literature that the optimal concentration range of SL for cryopreservation is 0.8% in dogs [20] to 1% in rams [14], humans [21] and cats [22] and goats [23]. The reason for this difference in addition of SL is that the differences in species are related to the differences of sperm plasma membrane composition between species [24]. The differences in the amount of concentration added might be due to different breeds of the rams, or different methods of preservation and different base liquor usage.

Our results showed that 0.5% concentration of SL dilution preserved sheep semen for 216 hours, had sperm progressive motility rate of 56.4%, the acrosome integrity rate of 75%, the pregnancy rate of 64.9%, and the lambing rate of 64.0%. Both were higher than the results revealed by Najafi et al [25] test, in which the sperm viability was 55.8% and membrane functionality was 44.5%, Mehdipour et al [26] test results showed that the viability of sperm was 41% and the membrane integrity was 49%, Forouzanfar et al [12] test results revealed that the sperm motility was 51.9%. The reason for these differences might be due to that when spermatozoa are preserved at low temperatures, cold blows must be prevented, which can in turn lead to irreversible sperm cells [27]. During the process of cryopreservation of spermatozoa, the process of freezing and thawing damages the ultrastructure of spermatozoa, leading to biochemical changes and loss of their vital content. The biochemical and ultrastructural changes of these spermatozoa under low temperatures might lead to a decrease of their functional integrity, survival rate and ability to bind to the ova [28]. The high concentration of SL has certain toxic effects on sperm motility and survival rate [12]. The addition of high concentration of SL in the diluent increased the viscosity of the diluent and particularly the debris in the diluent reduced the fertility of sperm together [29]. This might be subsequently related to the osmotic pressure of the diluent, decreasing with increasing concentration of SL in the diluent [30].

In conclusion, SL can successfully replace egg yolk as a supplement for preservation of sheep semen, without any adverse effects on sperm progressive motility, acrosome integrity, pregnancy rate, and lambing rate. Furthermore, our results showed that optimal lecithin concentration in the semen extender was 0.5% for preserving the ram semen. More investigations are warranted to accomplish evaluation of *in vitro* fertilizing ability of ram semen in the extender containing these concentrations of SL.

To our knowledge, this is the first report on the birth of lambs after the ewes were inseminated with ram semen stored for 216 hours. The ram sperm are capable of fertilization and producing normal offspring after preservation at 0°C with 0.5% of SL in Tris-based extender substituting for egg yolk.

## Figures and Tables

**Table 1:** Progressive motility of Duolang rams’ sperm stored at 0°C in diluents with different levels of soybean lecithin (mean±standard error of mean)

Groups	0 h	24 h	72 h	144 h	216 h	288 h
Control	91.9±4.9	86.7±4.2	76.4±6.4	66.1±3.2	56.2±3.2^a^	49.3±3.7^a^
SL (0.25%)	88.6±5.4	85.9±4.4	74.6±5.0	59.6±2.8	47.3±3.5^b^	38.1±5.0^b^
SL (0.5%)	88.4±3.7	84.9±3.4	74.8±2.9	66.4±3.3	56.4±2.6^a^	48.3±2.9^ab^
SL (0.75%)	90.5±2.6	84.7±3.9	72.2±1.3	65.1±1.4	54.6±3.0^ab^	46.8±3.8^ab^
SL (1.0%)	90.8±5.5	83.3±4.1	69.9±2.1	64.8±2.6	53.0±3.0^ab^	46.6±2.7^ab^
SL (1.25%)	89.2±5.6	83.4±4.6	75.7±4.6	64.3±3.1	53.3±2.7^ab^	46.4±5.4^ab^

Control, Tris-fructose egg yolk extender; SL, soybean lecithin.

a,b Values in the same column with different superscript differ (p<0.05).

**Table 2:** Acrosome integrity sperm of Duolang rams’ sperm in diluents supplemented with different levels of soybean lecithin (mean±standard error of mean)

Groups	0 h	24 h	72 h	144 h	216 h	288 h
Control	92.0±3.6	88.7±4.9	87.0±4.4	85.0±4.6	77.0±2.6	71.3±2.1
SL (0.25%)	89.7±4.5	87.7±3.5	85.3±4.2	83.0±5.3	77.7±4.5	70.3±5.5
SL (0.5%)	89.3±6.1	87.3±6.8	86.0±7.9	82.0±4.4	75.0±4.4	69.0±3.6
SL (0.75%)	89.7±7.1	87.0±6.1	84.0±5.3	80.0±5.0	72.0±1.7	67.3±2.1
SL (1.0%	90.7±4.5	86.3±4.5	84.7±4.2	81.3±4.7	73.0±1.0	66.7±2.5
SL (1.25%)	88.0±6.0	85.0±7.0	84.0±6.1	83.3±5.8	73.0±2.6	66.3±1.5

Control, Tris-fructose egg yolk extender; SL, soybean lecithin.

Values are not significant (p>0.05).

**Table 3:** Effect of the presence of soybean lecithin in the extender on *in vivo* results after artificial insemination of semen preserved for 216 h

Groups	Total number of inseminated ewes	Pregnancy rate (%)	Lambing rate (%)
Control	112	74(66.1)	71(63.4)
SL (0.5%)	111	72(64.9)	71(64.0)

Control, Tris-fructose egg yolk extender; SL, soybean lecithin.

Values are net significant (p>0.05).
